# The effects of a single session of chiropractic care on strength, cortical drive, and spinal excitability in stroke patients

**DOI:** 10.1038/s41598-019-39577-5

**Published:** 2019-02-25

**Authors:** Kelly Holt, Imran Khan Niazi, Rasmus Wiberg Nedergaard, Jens Duehr, Imran Amjad, Muhammad Shafique, Muhammad Nabeel Anwar, Harrison Ndetan, Kemal S. Turker, Heidi Haavik

**Affiliations:** 1 Centre for Chiropractic Research, New Zealand College of Chiropractic, Auckland, New Zealand; 2 Health & Rehabilitation Research Institute, Auckland University of Technology, Auckland, New Zealand; 3 SMI, Department of Health Science and Technology, Aalborg University, Aalborg, Denmark; 4 Riphah International University, Islamabad, Pakistan; 5 National University of Science and Technology, Islamabad, Pakistan; 6 University of North Texas Health Science Center, Tylers, Texas USA; 7 School of Medicine, Koç University, Istanbul, Turkey

## Abstract

The objective of this study was to investigate whether a single session of chiropractic care could increase strength in weak plantar flexor muscles in chronic stroke patients. Maximum voluntary contractions (strength) of the plantar flexors, soleus evoked V-waves (cortical drive), and H-reflexes were recorded in 12 chronic stroke patients, with plantar flexor muscle weakness, using a randomized controlled crossover design. Outcomes were assessed pre and post a chiropractic care intervention and a passive movement control. Repeated measures ANOVA was used to asses within and between group differences. Significance was set at p < 0.05. Following the chiropractic care intervention there was a significant increase in strength (F (1,11) = 14.49, p = 0.002; avg 64.2 ± 77.7%) and V-wave/Mmax ratio (F(1,11) = 9.67, p = 0.009; avg 54.0 ± 65.2%) compared to the control intervention. There was a significant strength decrease of 26.4 ± 15.5% (p = 0.001) after the control intervention. There were no other significant differences. Plantar flexor muscle strength increased in chronic stroke patients after a single session of chiropractic care. An increase in V-wave amplitude combined with no significant changes in H-reflex parameters suggests this increased strength is likely modulated at a supraspinal level. Further research is required to investigate the longer term and potential functional effects of chiropractic care in stroke recovery.

## Introduction

Stroke is one of the leading causes of death and disability in the world^1^. It is estimated that 17 million people per year suffer from a significant stroke worldwide, with 5 million of those people experiencing long term physical disability following the stroke^2^. The global burden of stroke continues to rise even though the rate of stroke related mortality has decreased over recent years^3^. Stroke often results in prolonged physical, emotional, social and financial consequences for stroke survivors, family, friends, and caregivers^2^. One of the most commonly occurring deficits associated with strokes is hemiparesis, which affects upper limb function, or an individual’s ability to stand, balance or walk^4^. The long term impaired nervous system function that accompanies many strokes means millions of stroke survivors are reliant on care-givers to assist them with rudimentary activities of daily living, such as bathing, dressing, and toileting^2^. The burden of care is immense and has a significant impact on modern society^2^.

This burden is even higher in developing countries. Disability-adjusted life years (DALYs), which is a measure of both years of life lost and years lived with a disability, is 7 times higher in developing countries compared to high income countries^5^. While part of this difference is possibly due to differences in age, incidence, and mortality in developing countries; the mortality of stroke has dropped by 20% while the age-standardized incidence of stroke increased by 12% between 1990 and 2010 in low and middle income countries^6^. This means more people have strokes, more survive, and as a result, the burden of stroke is even greater in developing countries.

Numerous rehabilitative approaches have been shown to promote motor recovery after a stroke^7–9^. These include physical therapy, motor re-learning, and brain computer interface-based approaches amongst others^7–9^. These approaches generally involve long-term treatment as a part of a large rehabilitation team^10^. Advanced strategies are constantly being developed and tested in an attempt to improve long term outcomes for stroke survivors^4^. One possible intervention that may improve post-stroke motor recovery, but has to date not been adequately tested, is chiropractic care. Chiropractic care involves an holistic approach to health with a particular focus on the relationship between the spine and nervous system^11^. Traditionally, the main focus of chiropractic care has been the location, analysis and correction of vertebral subluxations^11^. Vertebral subluxations are recognized as a biomechanical lesion of the spine by the World Health Organization (ICD-10-CM code M99.1)^12^. They have been defined as a self- perpetuating, central segmental motor control problem that involves a joint, such as a vertebral motion segment, that is not moving appropriately, resulting in ongoing maladaptive neural plastic changes that interfere with the central nervous system’s ability to self-regulate, self-organize, adapt, repair and heal^13^. Chiropractors identify vertebral subluxations using a combination of pathophysiologic indicators of spinal dysfunction^14^ and then correct them using a variety of manual techniques^15^, the most common being specific high-velocity, low amplitude adjustments that are delivered by hand to the subluxated spinal segment^16^.

Over the past two decades numerous research studies have shown that chiropractic care can significantly influence central neural function^17–19^. It has been hypothesized that the central neural plastic changes that are observed following chiropractic care may be due to improvements in spinal function associated with the correction of vertebral subluxations^20^. Studies have shown changes in somatosensory processing, sensorimotor integration and motor control following as little as a single session of chiropractic care^17,18,21–28^. Sensorimotor integration is the ability of the central nervous system (CNS) to integrate sensory information from different body parts and formulate appropriate motor outputs to muscles^29^. Effective sensorimotor integration is essential when learning new motor skills, or recovering from an injury^30,31^. Another essential component for accurate movement, learning new motor skills, and/or recovering from an injury, is the accuracy of internal representations of our body map, or body schema^32–34^. It is essential for our brain to be accurately aware of the location of our limbs and core body in 3D space^32^. The spine is linked biomechanically and neurologically to the limbs and yet, we know very little about how altered sensory feedback from the spine affects limb sensorimotor integration and motor performance. However, there is emerging evidence that altered spinal sensory input can alter central neural processing^21,35^, possibly by impacting the brains inner body schema. There is also emerging evidence that improving spinal function with chiropractic care can rapidly alter central neural function in a variety of ways^17,18,21–28^, and that these changes outlast the altered changes of input, i.e. that they are neural plastic changes.

If chiropractic care results in improvements in spinal function that have a central neural plastic effect, this may be important for a variety of clinical populations. Recently, Niazi, Turker^18^ reported an increase in plantar flexor muscle strength of 16% in reasonably healthy participants following a single session of chiropractic care. These authors also assessed possible neural plastic changes associated with chiropractic care by assessing the Hoffman reflex (H-reflex) and volitional waves (V-waves). By also assessing these reflexes it helped to establish whether changes in strength following chiropractic care were due to spinal or supraspinal influences^18^. The H-reflex and V-waves are neurophysiological measures that have previously been shown to change following chiropractic care and are also important indicators of changes in central nervous system function that are important for motor recovery following a stroke^18,36,37^. The H-reflex is largely modulated by presynaptic inhibition and motoneuron excitability (spinal input)^38^, and the V-wave is a measure of supraspinal input, or cortical drive, to the motor neuron pool^39,40^. Niazi, Turker^18^ reported changes in both the H- reflex and V-waves associated with increases in strength following a single session of chiropractic care in their study. These changes were similar to those observed following 3 weeks of strength training^40^.

Other small studies have also shown an increase in strength following a single session of chiropractic care^41–43^. Christiansen, Niazi^41^ found a significant 8% increase in plantar flexor muscle strength in elite taekwondo athletes after a single session of chiropractic care. One previous controlled pilot study reported a significant increase in quadriceps muscle strength following a single chiropractic adjustment session^42^. However, group allocation was not randomized, which resulted in baseline group differences, and post-intervention between group differences were not significant. Suter, McMorland^43^ also reported a decrease in quadriceps muscle inhibition and increased quadriceps muscle activation following a chiropractic adjustment session. If these changes are lasting, and also occur in people who have suffered from a stroke, they may be important for stroke recovery. The primary objective of this study was to investigate whether a single session of chiropractic care increased strength in weak plantar flexor muscles in chronic stroke patients. The secondary objective was to investigate at what level in the nervous system potential changes in strength were modulated.

## Methods

### Design and setting

This study was a randomized controlled crossover trial, with a minimum of 7 days between study sessions, that was conducted at Railway General Hospital in Rawalpindi, Pakistan. Railway General Hospital is a teaching hospital run by Riphah International University, Islamabad, Pakistan, and provides a broad range of medical services to inpatients and out- patients from the surrounding regions. Data were collected by a team of researchers from the Centre for Chiropractic Research at the New Zealand College of Chiropractic from April to June 2016. The trial was approved by the Research Committee at the New Zealand College of Chiropractic, all the participants gave written informed consent, which conformed to the Declaration of Helsinki and the study was approved by it the Riphah International University Research Ethics Committee in Pakistan (ref. # Riphah/RCRS/REC/000118). As a small basic science study this trial was not originally registered with a clinical trials registry. Following consultation, the study was registered retrospectively and was approved on 17^th^ June 2016 by the Australian New Zealand Clinical Trials Registry (Registration Number ACTRN12616000791437).

### Participants

To be eligible to participate in this study volunteers must have suffered from a stroke at least 12 weeks prior to their involvement in the trial and have ongoing plantar flexor muscle weakness, but have the ability to contract their plantar flexor muscles on command. Volunteers were drawn from the database of patients who had completed post-stroke rehabilitation at the Department of Physiotherapy and Rehabilitation at Railway General Hospital. Volunteers were ineligible to participate if they exhibited no evidence of spinal dysfunction (presence of vertebral subluxation indicators identified by a chiropractor), had absolute contraindications to spinal adjustments (including spinal fracture, atlanto-axial instability, spinal infection, spinal tumor, or cauda equina syndrome), or had experienced a previous significant adverse reaction to chiropractic care (defined as an untoward occurrence that results in death or is life threatening, requires hospital admission, or results in significant or permanent disability^44^).

### Experimental procedure

**Figure 1:**
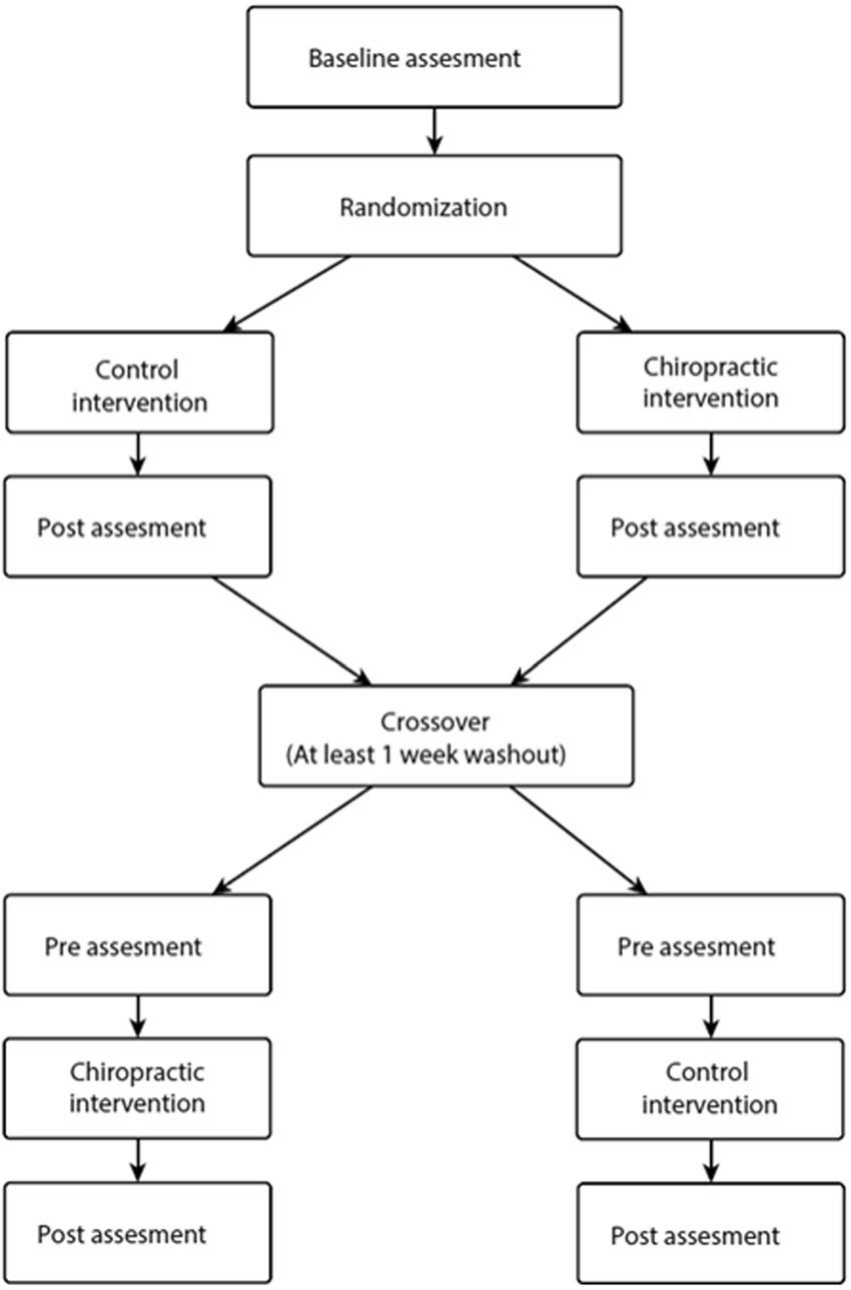
Flowchart of study flow.

Volunteers were assessed for eligibility criteria, and if eligible, they were asked to provide informed consent to participate in the trial. A baseline evaluation was then completed prior to group allocation. The appropriate intervention was then applied and immediately post the intervention the participant was reassessed using the same outcome measurement procedures. The participants were then reassessed after a minimum 7-day washout period with the alternate intervention applied between pre/post assessments. (See Fig. 1 for a diagram of the study flow).

### Interventions

The study involved a chiropractic care intervention and a passive movement control intervention.

#### Chiropractic Intervention

The entire spine and both sacroiliac joints were assessed for vertebral subluxations, and chiropractic adjustments were given where deemed necessary, by a New Zealand registered chiropractor. The clinical indicators that were used to assess for vertebral subluxations prior to and after each chiropractic care session included assessing for tenderness to palpation of the relevant joints, manually palpating for restricted intersegmental range of motion, assessing for palpable asymmetric intervertebral muscle tension, and any abnormal or blocked joint play and end-feel of the joints. These biomechanical and neurological characteristics are used in combination by chiropractors as clinical indicators of vertebral subluxations^14,45^. Using these indicators in a multidimensional battery of tests has been shown to be reliable for the identification of vertebral subluxations^45^, but the clinical validity of some of these tests remains unclear^14^. The chiropractic adjustments performed in this study were either high-velocity, low-amplitude thrusts to the spine or pelvic joints or instrument assisted adjustments^15^. These are standard adjustment techniques used by chiropractors^15^. These adjustment techniques have also previously been used in studies that have investigated the neurophysiological effects of chiropractic care^17^.

#### Control intervention

The control intervention involved the chiropractor performing a similar examination to the chiropractic care intervention followed by the participant being moved into adjustment setup positions similar to the chiropractic care intervention. The chiropractor did not contact on a segment deemed to be subluxated during the control set-up and no adjustive thrusts were applied during any control intervention. This control intervention was primarily intended to act as a physiological control for possible changes occurring due to the cutaneous, muscular or vestibular input that would occur with the type of passive and active movements involved in preparing a participant/patient for a chiropractic adjustment.

### Outcomes

Outcomes were assessed in the lower limb exhibiting the most muscle weakness immediately pre and post chiropractic care sessions and pre and post control intervention sessions. The outcomes assessed were change in absolute maximum force of contraction of the ankle plantar flexors (strength), change in the H-reflex (a measure of spinal excitability)^46^, and change in V-wave amplitude (a measure of cortical drive)^47^.

#### Absolute maximum force of contraction (strength)

Maximum isometric plantarflexion force was measured using an isometric strain gauge (Model MLP100 Transducer Techniques, Temecula, California, USA) mounted on a custom-built platform. The participants performed 3 progressive maximum voluntary contractions (MVCs) of the ankle plantar flexors of 5 seconds duration each, separated by a minimum of 2-minutes rest. Participants were verbally encouraged to produce maximal force. The largest of the MVCs measured in each experimental or control session was used for analysis and to compute the submaximal target contraction levels for H-reflex recruitment curve recordings.

#### H-reflex recruitment curve (spinal excitability)

During the H-reflex and direct motor response (M-wave) recruitment curve recording the participant was instructed to perform a plantarflexion of their affected leg equivalent to 10% of their MVC. The participant had a visual guide of their recorded force output to help maintain the 10% contraction level. While the participant performed this low-level contraction the M-wave and H -reflex were elicited via electrical stimulation of the tibial nerve. Electrical stimulations between the maximum M-wave stimulation intensity and zero were divided into 16 equally spaced steps. The participant was stimulated randomly with a varying time interval between 2 and 3 seconds at each intensity step 3 times. Evoked potential peak to peak amplitudes of the H-reflex were calculated offline from unrectified EMG (electromyography) signals. The H-reflex was normalized to the corresponding maximum M-wave. This was done since the size of the M-wave is affected by contraction intensity^48^. Furthermore, the M-waves were fitted so that pre and post M-wave curves could be superimposed on top of each other allowing any genuine change in the H-reflex curve to be highlighted. This was done based on the method described by Brinkworth, Tuncer^49^. The H-reflex recruitment curve was fit by a general least squares model described by Klimstra and Zehr^46^ and the following parameters were analyzed: stimulation level of the H-threshold, level at 50% of Hmax (s50), and the slope of the ascending limb of the H-reflex. Changes in these H-reflex parameters reflect changes in neuromodulation at the level of the spinal cord^38,46^.

#### V-wave (cortical drive)

The participant performed 3 MVCs of 5 seconds duration with a 2-minute rest period between MVCs. During the MVCs, 3 supramaximal stimuli (110% of the current needed to evoke the maximal M-wave) were applied to the tibial nerve when the force recorded was 90% or above the level recorded during MVC. V-wave peak to peak amplitude was calculated using similar methods to those used with the H-reflex and were also normalized to the corresponding maximum M-wave. V-wave peak to peak amplitude, normalized to the maximum M-wave (V-wave/Mmax ratio), was the outcome measure used for analysis and reflects cortical neural drive to spinal motoneurons^47^.

### Instrumentation

All tests were performed on the participants weakest leg with the participant comfortably seated in a chair and their weakest leg placed in an isotonic force transducer (OT Bioelettronica EMG-USB2+). Their weakest leg was firmly strapped to the bottom plate of the force transducer. Bipolar surface electrodes (20 mm Blue Sensor Ag/AgCl, AMBU A/S, Denmark) were used to record surface electromyography (EMG) activity from the affected soleus muscle (SOL). The electrodes were placed 2 cm apart on the belly of the muscle, and a grounding band was placed on the corresponding ankle. Before the placement of the electrodes, the skin was shaved, if needed, slightly abraded and cleaned with alcohol. The EMG signals were amplified by a factor of 1000 using a bipolar amplifier (OT Bioelettronica). The signal was recorded with a CED Power 1401 MK 2 data acquisition board at 5 kHz and to measure the H-reflex, data were digitally band-pass filtered between 20–1000 Hz.

The H-reflex, M-waves, and V-waves of the SOL were elicited by stimulation of the tibial nerve. The electrical stimulation was delivered using an isolated single pulse stimulator (Digitimer DS7AH, UK). The stimulating electrodes (PALS RECT 5 × 9 cm, cathode) were placed proximal to the patella and in the popliteal fossa (PALS RND 3.2 cm, anode). The stimulation intensity and placement of the anode was manipulated until the greatest response with the minimal stimulus intensity was achieved.

### Sample size

Sample size calculations were based on detecting a difference in a continuous response variable between control and experimental intervention sessions. Calculations were performed using G-Power 3.1 software based on the changes observed in a previous study that investigated changes in force in plantar flexor muscle strength pre and post a chiropractic care session^18^. If the true difference in mean MVC between the experimental session and the control session had an effect size of 0.5 it was calculated that 12 participants would be required to reject the null hypothesis that the changes in population means of the experimental and control groups were equal with probability (power) 0.8. The Type I error probability associated with the test of this null hypothesis is 0.05. To allow for the relative uncertainty relating to power outcomes due to the study being conducted in a stroke population, we aimed to enroll 15 participants in this trial.

### Randomization and blinding

Allocation of participants to order of intervention was carried out using an online randomization program. The randomization sequence was created using Minimizer (Microsoft Corp., Redmond, WA) with a 1:1 allocation to chiropractic care intervention or control intervention first. Participants were not informed which group they were in during the study.

Chiropractors providing care were unable to be blinded to intervention allocation. Participants were not informed which group they were in during each session. Participants were naïve to chiropractic care so may not have been aware which intervention they were receiving, but this cannot be confirmed. All recorded data were anonymized and coded prior to analysis and the data analyst and independent statistician remained blind to group allocation during the analysis period.

### Statistical analysis

Two-way repeated measures analysis of variance (ANOVA) was used to assess for differences in MVC’s of the plantar flexors, soleus evoked V-waves, and H-reflex parameters before and after the 2 interventions. Time (pre and post intervention measures) and Intervention (chiropractic vs control) were used as factors. Post hoc pairwise comparisons were made using Tukey’s HSD tests if required. Statistical significance was set at p < 0.05 for all comparisons. Percentage change for each measure was computed subject-wise, using the following formula (post − pre)/pre × 100.

## Results

### Participants and baseline outcome measures

We aimed to enrol 15 patients in this trial, but only 12 could be recruited in the time available. All participants were male (age 49 ± 8 years), who had all suffered a stroke affecting motor control of their lower leg (10 middle cerebral artery and 2 anterior cerebral artery strokes). The time since stroke varied between 3 and 36 months (13.7 ± 11.2 months) (See Table 1 and participant flow in supplementary file).

**Table 1:** Clinical characteristics of patients.

Variable	n = 12
**Gender**	
Male	12
Age	48 ± 7
**Stroke location (all occlusive)**	
Middle cerebral artery	10
Anterior cerebral artery	2
**Affected side**	
Left	4
Right	8
**Time Since Stroke (Months)**	
Average	14 ± 11.7
Range	3–36

Table 2 shows baseline outcome measures at each assessment. There were small differences in baseline strength and V-wave/Mmax ratio between assessments, but these differences didn’t approach significance and they were not influenced by the order of intervention. Therefore, there was unlikely to be a long-term effect of the chiropractic care intervention that could have influenced the second assessment in the ‘chiropractic care first’ group.

**Table 2:** Baseline and Post Intervention (Mean ± SD) values for Force, V-wave/Mmax ratio and H-reflex parameters.

Parameters	Chiropractic (Mean ± SD)			Control (Mean ± SD)			(Intervention * Time) P value
	Pre	Post	% change	Pre	Post	% change	
Absolute Force (Kgs)	15.2 ± 8.2	23.5 ± 13.7	64.2 ± 77.7	18.8 ± 9.9	13.4 ± 7.3	−26.4 ± 15.5	P = 0.002
V-wave/Mmax ratio	8.0 ± 4.4	11.8 ± 6.9	54.0 ± 65.2	12.7 ± 9.8	10.6 ± 6.8	−12.1 ± 13.8	P = 0.009
H-Threshold (Intensity step)	3.6 ± 1.2	3.8 ± 1.6	10.4 ± 1.0	3.3 ± 1.2	3.7 ± 1.8	15.1 ± 48.5	P = 0.35
s50 (Intensity step)	10.4 ± 1.0	9.8 ± 1.2	−6.0 ± 8.7	10.3 ± 1.9	10.0 ± 2.1	−2.8 ± 11.8	
Slope	1.0 ± 0.5	1.3 ± 0.7	23.0 ± 39.8	1.1 ± 0.7	1.1 ± 0.7	2.4 ± 25.4	

### Chiropractic care

All participants received at least 1 chiropractic adjustment in their cervical, thoracic and lumbopelvic spinal regions. A combination of chiropractic technique approaches were used on most participants; including high velocity low amplitude adjustments and instrument assisted adjustments. If vertebral subluxation indicators did not change at a specific level following an adjustive thrust a second thrust or alternate technique was used when deemed appropriate. Due to the combination of adjustment levels and techniques that were used, no conclusions can be made from this study about the influence that specific adjustment sites or techniques had on patient outcomes.

### MVC Force

**Figure 2:**
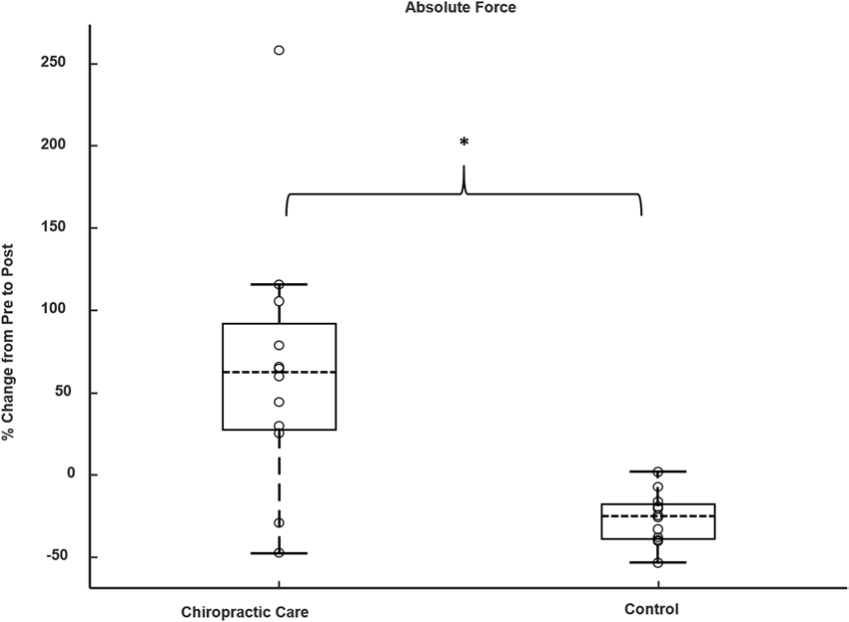
Boxplot for percentage change from the pre-interventions for absolute force (N = 12), ‘o’ represents an individual data point, ‘------’ represents the median value, *p < 0.05.

There was a significant increase in plantarflexion MVC force after chiropractic care compared to the control intervention (p = 0.002). Following chiropractic care, on average, force increased by 64.2 ± 77.7% (p = 0.02) and a decrease of 26.5 ± 15.5% (p = 0.001) was observed following the control intervention. MVC results are presented in Fig. 2.

### H-Reflex

**Figure 3:**
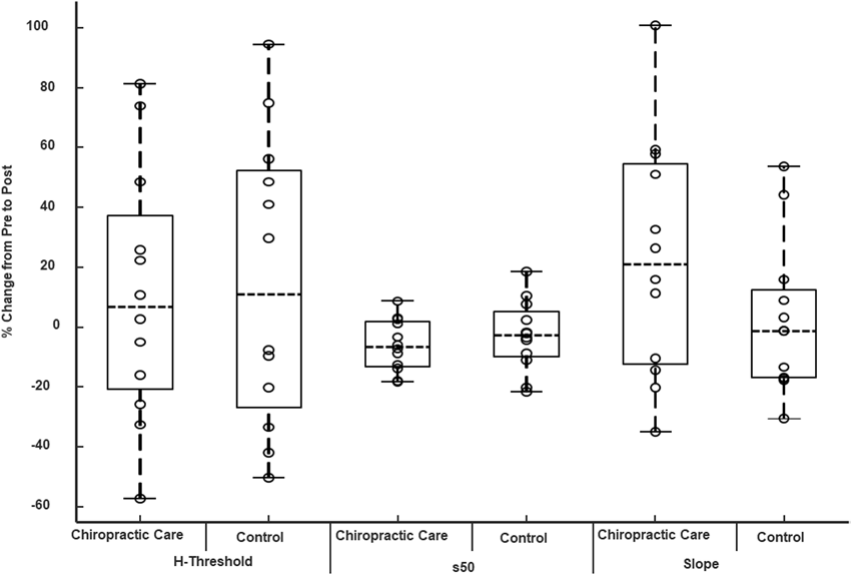
Effect of chiropractic care on H-reflex parameters: the effects are shown in the boxplot for percentage change from the values obtained from the period preceding the chiropractic care or control intervention. ‘o’ represents an individual data point, ‘------’ represents the median value There were no significant changes in the threshold for eliciting the reflexes, s50 or slope following either intervention.

Following the chiropractic intervention, the H-reflex threshold decreased, the H-reflex threshold level at 50% of Hmax (s50) decreased, and the slope of the ascending H-reflex became steeper compared to the control intervention. However, none of these changes were statistically significant. The H-reflex parameter results are presented in Table 2 and illustrated in Fig. 3.

### V-Wave

**Figure 4:**
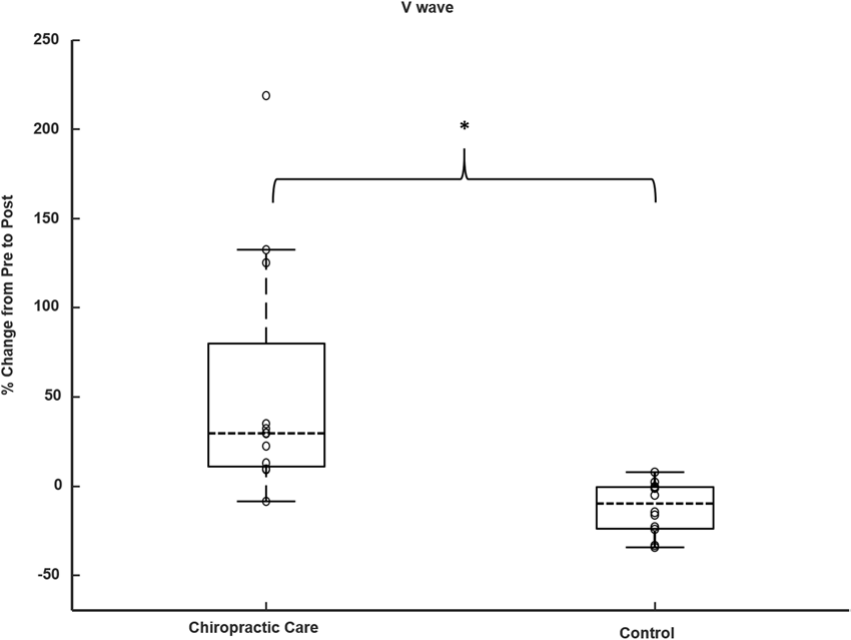
Boxplot for percentage change from the pre-interventions for V-wave/Mmax ratio (N = 12), ‘o’ represents an individual data point, ‘------’ represents the median value, *p < 0.05.

There was a significant increase in V-wave/Mmax ratio after chiropractic care compared to the control intervention (p = 0.009). The V-wave/Mmax ratio increased by 54.0 ± 65.2% (p = 0.03) after the chiropractic care session and a non-significant decrease in V-wave/Mmax ratio occurred after the control intervention (−12.1 ± 13.8%, p = 0.07). See Fig. 4.

## Discussion

### Summary of main findings

The key findings in this study were that in a group of chronic stroke patients, with lower limb muscle weakness, plantarflexion muscle strength increased on average by 64.2% following a chiropractic care session and the change in muscle strength appears to be modulated by cortical factors as opposed to modulation at the spinal level.

### Compared with the Literature

A similar approach to the current study was taken in a previous study. Niazi, Turker^18^ investigated the effect of a single session of chiropractic care on plantar flexor muscle strength, the H-reflex, and V-waves in subclinical pain patients using a crossover study design. They reported that following chiropractic care there was an increase in strength of 16.05 ± 6.14% (p < 0.01), an increased V-wave/Mmax ratio of 44.97 ± 36.02% (p < 0.01) and reduced H-reflex threshold. Following the control intervention strength reduced (fatigued) by 11.35 ± 9.99% (p = 0.03) and the V/Mmax ratio decreased by 23.45 ± 17.65% (p = 0.03). They concluded that chiropractic adjustments appear to alter the net excitability of low-threshold motor units and increase cortical drive, which may explain why the increase in strength that they observed occurred. They also suggested that chiropractic adjustments may prevent fatigue. In the present study, significant changes were also observed in strength and V-wave/Mmax ratio results, however the changes in strength were far greater in the present study. The greater percentage increase in strength in the present study may be due to the stroke patients having weaker muscles to begin with, so they had more opportunity to increase in strength. Following the control session in the present study the reduction in V-wave/Mmax ratio also failed to reach statistical significance (p = 0.07), so no conclusions can be made about a potential reduction in fatigue based on this finding. The biggest difference between these 2 studies was that in the present study no significant difference in H-reflex parameters were observed, which may indicate that the changes in strength in the present study were more heavily influenced by an increase in cortical drive as opposed to spinal excitability.

Christiansen, Niazi^41^ also followed a similar study protocol to the present study in their controlled crossover trial that investigated the effects of a single session of chiropractic care on strength and cortical drive in elite taekwondo athletes. They reported similar findings to the present study with a significant increase in strength and cortical drive following chiropractic care, and a significant decrease in strength and cortical drive following the control intervention. They reported potential trends in changes to H-reflex parameters following chiropractic adjustments, but these results failed to reach significance. The results reported by Christiansen, Niazi^41^ are congruent with those from the present study and suggest that the changes in strength observed appear to be more likely due to changes in cortical drive instead of modulation of spinal cord excitability. Interestingly, Christiansen, Niazi^41^ followed their participants for 60 minutes post intervention and found that changes in strength were still significant 30 minutes post intervention but they failed to meet significance at the 60 minute evaluation. However, changes in the V-wave/Mmax ratio persisted until at least 60 minutes post intervention.

Many studies have investigated a variety of interventions to help promote motor recovery after stroke. Successful interventions generally involve long term rehabilitative training over several weeks or months that include interventions such as progressive resistance training, specific task training, functional electrical stimulation, aerobic cycling, and robot- assisted therapy^7–9^. However, the current study is the first study to investigate changes in strength in stroke patients following chiropractic care or spinal manipulation.

### Possible mechanisms

Facilitation and modulation of neural plasticity is thought to be the key to promoting motor recovery in stroke patients^50^. Recovery of strength following stroke is mainly due to cortical plastic reorganization in the early phases of rehabilitation^50^. Changes in maladaptive neural plasticity are also important in stroke recovery, particularly when it comes to spasticity. It has been hypothesized that vertebral subluxations are central segmental motor control problems that result in ongoing maladaptive neural plastic changes in the central nervous system^13,17,23,27^. Central neural plastic changes have been observed following chiropractic care which may be due to improvements in spinal function associated with the correction of vertebral subluxations^20^. It is therefore possible that the improvements in muscle strength following chiropractic care observed in this study were due to changes in maladaptive neural plasticity that resulted in increased descending drive to the leg muscles.

Strong placebo effects have also been hypothesized to occur following chiropractic care due to manual contact, care provider attention, and provider enthusiasm^51,52^. In the present study the care providers had limited communication with study participants due to language barriers and participants were naïve to chiropractic care so may not have been aware which intervention they were receiving. However, it is unclear whether participants were aware if they had received the experimental or control intervention, so it is possible that participants simply tried harder following the chiropractic care intervention.

### Clinical and research implications

This study involved a small subgroup of stroke patients and a single chiropractic care session, and thus represents a basic science study exploring mechanisms as opposed to a clinical trial investigating efficacy. It is unclear how long the strength changes observed in this study lasted, and whether they had an impact on functional ability. This would need to be explored in future studies as stroke rehabilitation is rarely evaluated based on short-term strength changes. It is also unclear whether longer term chiropractic care may result in beneficial functional changes in motor control in stroke patients. This would also require a follow up randomized clinical trial. Finally, it is unclear whether the changes observed in this small group of male stroke patients from Pakistan are generalizable to other stroke populations. Further research is now required to investigate whether longer term chiropractic care has clinically significant benefits in strength and functional ability in stroke patients. Future clinical trials should use a parallel group design, should include long term care interventions, include clinically relevant outcomes, and consider including an active control intervention.

### Strengths and limitations

Limitations of this study include that it had a small sample size (n = 12), and recruitment targets were not met (n = 15). However, significant results were observed in strength and V-wave/Mmax ratio outcomes, which suggests it was adequately powered for the primary outcome measure. The small sample size may have meant that type II errors occurred in the V-wave/Mmax ratio results for the control intervention and the H-reflex findings.

Trials such as this impede effective blinding of participants and care-givers due to the nature of the chiropractic intervention^53^. The present study was somewhat unique with respect to participants being naïve to chiropractic care, but the possibility of placebo or Hawthorn effects following the chiropractic intervention does exist. Future trials could assess participant blinding using a questionnaire to investigate this limitation.

The crossover nature of the trial design is a strength of the study, as the participants act as their own control. However, it is unclear how long the effects of the chiropractic intervention last, which may mean the washout period of 7 days was inadequate. Although the order of intervention did not significantly alter pre-assessment outcomes it is possible that residual effects of the chiropractic intervention were still present after 7 days.

## Conclusion

In this group of stroke patients, with plantar flexor muscle weakness, a single session of chiropractic care resulted in increased plantar flexor muscle strength and cortical drive to the affected limb. Further research is required to investigate the longer term and potential functional effects of chiropractic care in stroke recovery.

## Supplementary information

consort check list

cosort flow diagram
