# Supplementary material for: The effects of a single session of chiropractic care on strength, cortical drive, and spinal excitability in stroke patients
Source: Sci Rep. 2019 Feb 25;9:2673. doi: 10.1038/s41598-019-39577-5 (PMC6389925; doi:10.1038/s41598-019-39577-5)
Supplement: Supplementary file 2 — cosort flow diagram [file 41598_2019_39577_MOESM2_ESM.doc]

**
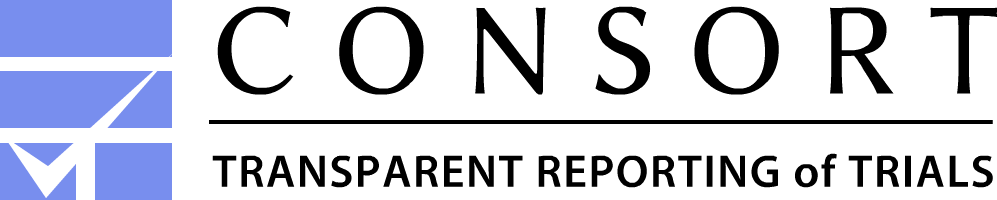
**

**CONSORT 2010 Flow Diagram**

**Allocation**

**Analysis**

**Follow-Up**

**Enrollment**

Assessed for eligibility (n=12)

Excluded (n=0)

Analysed (n=7)

Lost to follow-up (n=0)

Allocated to chiropractic intervention first (n=7)

♦ Received allocated intervention (n=7)

Lost to follow-up (n=0)

Allocated to control intervention first (n=5)

♦ Received allocated intervention (n=5)

Analysed (n=5)

Randomized (n=12)
